# Real‐World Outcomes With Low‐Dose Dasatinib (50 mg) in Imatinib‐Resistant Chronic Myeloid Leukemia in Chronic Phase: A Retrospective Analysis of Efficacy and Safety

**DOI:** 10.1002/cnr2.70400

**Published:** 2026-01-09

**Authors:** Nandhini Gangadaran, Harshal Mamlekar, Souvik Saha, Rajesh Kashyap, Sanjeev Yadav, Khaliqur Rahman, Ruchi Gupta, Mona Vijayaran, Manish Singh, Dinesh Chandra

**Affiliations:** ^1^ Department of Hematology Sanjay Gandhi Post Graduate Institute of Medical Sciences Lucknow Uttar Pradesh India

**Keywords:** BCR‐ABL positive chronic myeloid leukemia, dasatinib, imatinib mesylate, pleural effusion, tyrosine kinase inhibitors

## Abstract

**Background:**

Dasatinib, a potent second‐generation tyrosine kinase inhibitor (TKI), is highly effective in chronic myeloid leukemia in chronic phase (CML‐CP) resistant to imatinib at standard dosing (100 mg daily), but is often limited by adverse events. Emerging evidence suggests low‐dose dasatinib (50 mg daily) may maintain efficacy with improved safety, but data in imatinib‐resistant CML‐CP remain limited.

**Aims:**

To evaluate the efficacy and safety of low‐dose dasatinib (50 mg daily) in patients with imatinib‐resistant CML‐CP and to identify predictors of treatment response and disease progression.

**Methods and Results:**

This retrospective cohort study included 53 adults with imatinib‐resistant CML‐CP treated with low‐dose dasatinib at a tertiary center in Northern India (2002–2025). Early molecular response (EMR), major molecular response (MMR), deep molecular response (DMR), progression‐free survival (PFS), overall survival (OS), and adverse events were assessed. Multivariate Cox regression identified predictors of poor response and disease progression. Among 53 patients (median age 50 years), 41.5% achieved MR4.5, 20.8% MR4.0, and 15.1% MMR without DMR. Prior loss of MMR on imatinib significantly correlated with a superior response to dasatinib (*p* = 0.002). TKD mutations were present in 32.1%; the T315I mutation, high ELTS risk, and baseline BCR‐ABL1 ⟩ 100% independently predicted poor response. Clinically significant adverse events occurred in 49.1%, primarily cytopenias and pleural effusion. Among our cohort, 22.6% required a TKI switch due to inadequate response and 7.5% due to intolerance.

**Conclusion:**

Low‐dose dasatinib is effective and tolerable in imatinib‐resistant CML‐CP, with nearly two‐thirds achieving DMRs. Predictive biomarkers (T315I mutation, high ELTS risk, high baseline BCR‐ABL1) can guide dose optimization.

## 1 Introduction

Chronic myeloid leukemia (CML) is characterized by the presence of the Philadelphia chromosome t(9;22) (q34;q11), resulting in the formation of the BCR‐ABL fusion gene, that drives the leukemic stem cell proliferation [1]. The incidence of CML is 1–2 cases per 100 000 adults [1], with a median age of diagnosis around 64 years [2]. Before the TKI (tyrosine kinase inhibitor) era, the treatment strategies included hydroxyurea, recombinant interferon‐alfa (rIFN‐α), and low‐dose cytarabine. These therapies achieved hematologic responses in almost all of the patients, with limited complete cytogenetic responses (~14.5%), often accompanied by significant toxicity [3]. Allogeneic hematopoietic cell transplantation (HCT), the only curative option, was limited to younger, fit patients because of its substantial treatment‐related mortality (20%–40%) and graft‐versus‐host disease risks [4].

The introduction of imatinib, the first generation TKI inhibitor, in 2001 revolutionized the treatment of CML, by specifically targeting BCR‐ABL1. In the pivotal IRIS trial, imatinib achieved an estimated rate of complete cytogenetic response of ~76.2%, and demonstrated a more favorable safety profile [3]. However, resistance to first line imatinib therapy develops in up to one‐third of patients, highlighting the need for alternative treatment strategies [5]. Dasatinib, a highly potent second‐generation TKI, is approved for the treatment of CML‐chronic phase (CML‐CP) patients who exhibit imatinib resistance, with a standard recommended dose of 100 mg once daily [6].

Dasatinib is about 325 times more potent than imatinib, against BCR‐ABL1 [6]. In the CA180‐034 phase 3 dose‐optimization study, dasatinib 100 mg once daily maintained efficacy with fewer adverse effects [7]. Seven‐year follow‐up of this study showed progression‐free survival (PFS) rates of 49% and overall survival (OS) rates of 71% in the imatinib‐resistant/‐intolerant patients with CML‐CP [8]. Pleural effusions and cytopenias remain a concern with standard dosing [9]. Emerging data suggest a 50 mg daily dose might offer comparable efficacy with improved tolerability [10, 11]. While some studies have explored low‐dose dasatinib (50 mg) as frontline therapy in newly diagnosed CML‐CP, its role in imatinib‐resistant/intolerant patients remains understudied and warrants thorough investigation.

This study evaluates real‐world outcomes of low‐dose dasatinib in a homogenous cohort of imatinib‐resistant patients with CML‐CP, managed in a high‐volume tertiary center, focusing on molecular responses, survival, toxicity management, and mutation‐driven resistance. The findings aim to enable optimization of dosage strategies for second‐line dasatinib therapy following imatinib failure.

### 1.1 Objectives

#### 1.1.1 Primary Objective

1. To correlate treatment outcomes with low‐dose dasatinib in imatinib‐resistant CML‐CP patients, with respect to the achievement of early molecular response (EMR), major molecular response (MMR), and deep molecular response (DMR).
2. To assess the safety profile of low‐dose dasatinib, including the incidence and severity of adverse events, particularly pleural effusions, cardiac events, cytopenias, and GI symptoms.

#### 1.1.2 Secondary Objectives

1. To analyze PFS and OS in imatinib‐resistant or ‐intolerant CML‐CP patients treated with low‐dose dasatinib.
2. To identify factors predicting optimal response to low‐dose dasatinib.
3. To correlate baseline BCR‐ABL levels and KD mutation status with treatment outcomes.

## 2 Methodology

This is a retrospective cohort study analyzing data from CML‐CP patients resistant to imatinib who were subsequently treated with low‐dose dasatinib, under the Department of Hematology, SGPGIMS, Lucknow. Patient data were extracted from medical records and the Hospital Information System (HIS) over a 23‐year period, spanning from July 2002 to April 2025. The study protocol was reviewed and approved by the Institutional Ethics Committee, ensuring adherence to ethical guidelines for retrospective data analysis and patient confidentiality. Eligible participants were adults (≥ 18 years) with CML‐CP, ECOG performance status 0–2, documented imatinib resistance per ELN 2024 criteria, and initiation of low‐dose dasatinib with available baseline BCR‐ABL transcript data (with or without kinase domain mutation analysis). Patients were excluded if they had accelerated or blast‐phase disease, prior second‐generation TKI therapy, significant cardiovascular disease, baseline pleural or pericardial effusion, pregnancy or breastfeeding, or insufficient follow‐up data.

Data were systematically extracted from patient medical records and the HIS. All patients diagnosed with CML‐CP were initially started on standard‐dose imatinib 400 mg OD. These patients were followed up periodically for hematological and molecular response with quantitative BCR‐ABL1 levels at 3, 6, and 12 months, and every 3 months thereafter. Patients who failed to achieve BCR‐ABL^IS^ < 10% (EMR) in 3 months, < 1% in 6 months, < 0.1% (MMR) in 12 months, or loss of MMR at any timepoint thereafter (ELN 2024 criteria) [12], were managed with a dose escalation of imatinib to 600 mg OD for 3 months. During the 23‐year study period (2002–2025), the definition of imatinib resistance was based on the ELN criteria applicable at the time of patient management, including earlier versions prior to the 2024 update. Dose escalation of imatinib, though no longer recommended in current guidelines, was a widely accepted strategy in the earlier part of the study period, especially in resource‐constrained settings where second‐generation TKIs were not yet affordable or accessible. The patients who still failed to respond were labeled as “Imatinib resistant” and were switched to low‐dose dasatinib (50 mg OD). Failure of response was confirmed in two consecutive samples taken not more than 2 weeks apart. Patients on dasatinib were followed up at 3‐month intervals and evaluated for clinical, hematological, and molecular response. Patients with prior MMR loss, who failed to regain MMR after 3 months were escalated to standard‐dose dasatinib (100 mg daily). Continued non‐responders were designated dasatinib‐resistant and offered nilotinib or ponatinib as available and appropriate. Tyrosine kinase domain (TKD) mutation analysis was performed by Sanger sequencing, for academic purposes in all the patients at variable timepoints after 2018, when the testing became available in our institute. Adverse events—pleural effusion, cytopenia, cardiac events, GI toxicity, and derangement of liver and renal functions—were monitored for and documented, at every visit and managed according to the severity.

The following information was collected for each CML‐CP patient using a standardized data collection form:

1. Demographic data: age, gender, date of CML diagnosis.
2. Baseline characteristics:
  (i) Hemoglobin (Hb), platelet count, and total leukocyte count (TLC) at the time of CML diagnosis.
  (ii) Quantitative BCR‐ABL1 transcript levels (IS) at baseline (i.e., prior to imatinib initiation).
  (iii) Quantitative BCR‐ABL1 transcript levels (IS) prior to dasatinib initiation.
  (iv) EUTOS long‐term survival scores (ELTS) were calculated based on available baseline data to assess the risk stratification of patients.
3. Treatment data:
  (i) Imatinib dose and duration of response, any dose adjustments made during the course of treatment.
  (ii) Dasatinib dose and duration of therapy.
  (iv) Dasatinib response, resistance, and switch to a different TKI (nilotinib/ponatinib).
4. Response assessment:
  (i) Hematologic response at various time points (3, 6, 12 months, and every 3 months thereafter) was recorded. Complete hematologic response (CHR) was defined as normalization of blood counts (WBC < 10 × 10^9^/L, neutrophils > 1.0 × 10^9^/L, platelets < 450 × 10^9^/L, and absence of blasts in peripheral blood) [13].
  (ii) Molecular response: Serial measurements of BCR‐ABL1 transcript levels (IS) were collected at 3, 6, 12 months, and every 3 months thereafter to monitor achievement of EMR, MMR, and DMR, which is MR4.0 (BCR‐ABL1 ≤ 0.01% IS) and MR4.5 (BCR‐ABL1 ≤ 0.0032% IS) [12].
  (iii) Results of kinase domain mutation analysis by Sanger sequencing.
5. Survival data:
  (i) PFS: defined as the time from dasatinib initiation to disease progression (transformation to accelerated/blast phase), loss of response, or death due to CML‐related causes.
  (ii) OS: defined as the time from diagnosis of disease to death from any cause.
6. Adverse events:
  (i) Information on all adverse events experienced during dasatinib therapy was collected, including the type, grade, and severity of the events (using the Common Terminology Criteria for Adverse Events [CTCAE] version 5.0).
  (ii) Particular attention was given to adverse events of interest, such as:
    ○ Cytopenias (anemia, neutropenia, thrombocytopenia)
    ○ Pleural effusion
    ○ Cardiovascular events (e.g., heart failure, arrhythmias)
    ○ Gastrointestinal toxicity (e.g., nausea, diarrhea)
    ○ Deranged liver function
    ○ Deranged renal function
  (iv) Management of adverse events: details on interventions used to manage adverse events, including dose adjustments (interruptions or reductions) and TKI switch, supportive care measures (e.g., transfusions, growth factors), and specific treatments (e.g., diuretics for pleural effusion) were recorded.

Extracted data were reviewed and validated by a second researcher to identify and correct any errors or inconsistencies. A total of ~53 patients with complete data were enrolled in the study retrospectively. The minimum period of follow‐up was ~16 months from diagnosis.

### 2.1 Statistical Analysis

Statistical analyses were performed using SPSS version

1. Descriptive statistics:
  (i) Continuous variables were summarized using mean ± standard deviation (SD) or median with interquartile range (IQR), as appropriate.
  (ii) Categorical variables were summarized using frequencies and percentages.
2. Response analysis:
  (i) Molecular response rates (EMR, MMR, DMR) were calculated as the proportion of patients achieving the specified response at the designated time points.
  (ii) Differences in response rates between subgroups were compared using the chi‐square test or Fisher's exact test, as appropriate.
3. Survival analysis: PFS and OS were estimated using the Kaplan–Meier method.
4. Predictive modeling:
  (i) Multivariate Cox proportional hazards regression analysis was used to identify factors predictive of poor response to low‐dose dasatinib as well as disease progression to accelerated/blast crisis. Variables included in the model were:
    ○ Age
    ○ Gender
    ○ Baseline BCR‐ABL1 transcript levels before starting imatinib
    ○ BCR‐ABL1 transcript levels before starting dasatinib
    ○ KD mutation status (presence/absence and specific mutation type such as T315I)
    ○ ELTS risk scores
    ○ Duration of response to imatinib
  (ii) Hazard ratios (HR) and 95% confidence intervals (CI) were calculated to quantify the association between each variable and the risk of poor response or disease progression.
5. Safety analysis:
  (i) The incidence and severity of adverse events were summarized using frequencies and percentages.

## 3 Results

### 3.1 Baseline Characteristics

The study included 53 imatinib‐resistant CML‐CP patients who were started on low‐dose dasatinib. The median age was 50 years, ranging from 42 years to 77 years. There were 27 (51%) males and 26 (49%) females. At the time of presentation, the median TLC is 148 × 10^9^/L (range: 55–426 × 10^9^/L); the median hemoglobin is 10.1 g% (range: 5.8–13.6 g%); the median platelet count is 259 × 10^9^/L (range: 72–871 × 10^9^/L). Of the 53 patients, the ELTS risk score was high in 22 cases (41.5%), intermediate in 15 (28.3%), and low in 16 (30.2%) cases. The median BCR‐ABL1 transcript level at baseline (at the time of diagnosis) was 69.4% IS. The median BCR‐ABL1 transcript levels before initiation of low‐dose dasatinib fell between 0.1% and 1% IS. Molecular characteristics of the study population at the time of initiation of low‐dose dasatinib are summarized in Table 1.

**TABLE 1:** Molecular characteristics of the study population at the time of initiation of low‐dose dasatinib (*n* = 53).

S. no.	Patient subgroup based on prior response to imatinib	*n* (%)	Median duration of response to imatinib in years (range in years)	BCR‐ABL1% at the time of dasatinib initiation (IS)
1	Lost MMR after initial achievement	34 (64.2%)	4.5 (1–10)	0.1–1
2	Lost MMR and EMR after initial achievement	5 (9.4%)	4.0 (1–12)	10–100
3	Achieved EMR but never achieved MMR	7 (13.2%)	0.5	1–10
4	Primary resistance—never achieved EMR	7 (13.2%)	0	10–100

### 3.2 Molecular Response to Low‐Dose Dasatinib

Of the 53 patients treated with low‐dose dasatinib, the responses across different patient subgroups were stratified as follows: 22 patients (41.5%) achieved MR4.5, 11 (20.8%) achieved MR4.0, 8 (15.1%) attained MMR without DMR, 5 (9.4%) achieved EMR without MMR, and 7 (13.2%) exhibited primary resistance, failing to achieve EMR. Table 2 depicts the correlation between the response of our study cohort to low‐dose dasatinib and patient subgroups based on prior response to imatinib.

**TABLE 2:** Correlation of response of study population to low‐dose dasatinib with patient subgroups based on prior response to imatinib.

S. no.	Patient subgroup based on prior response to imatinib	*n* (%)	Response to low‐dose dasatinib	Median duration of follow‐up in months (range)	*p*
1	Lost MMR after initial achievement (*n* = 34, 100%)	21 (61.8%)	Achieved DMR 4.5	51 (2–127)	0.002
		7 (20.6%)	Achieved DMR 4.0	58 (14–223)	
		6 (17.6%)	Regained MMR, but not DMR	32 (12–169)	
2	Lost MMR and EMR after initial achievement (*n* = 5, 100%)	1 (20%)	Achieved DMR 4.0	23	0.78
		1 (20%)	Regained MMR, but not DMR	38	
		3 (60%)	Never regained EMR	69 (44–119)	
3	Achieved EMR but never achieved MMR (*n* = 7, 100%)	1 (14.3%)	Achieved DMR 4.0	24	0.18
		1 (14.3%)	Achieved MMR, but not DMR	45	
		5 (71.4%)	Maintained EMR, but never achieved MMR^a^	72 (25–124)	
4	Primary resistance—never achieved EMR (*n* = 7, 100%)	1 (14.3%)	Achieved DMR 4.5	24	0.35
		2 (28.6%)	Achieved DMR 4.0	44.5 (23–66)	
		4 (57.1%)	Never achieved EMR	64.5 (35–146)	

^a^ A distinct subgroup maintained the EMR (BCR‐ABL1 IS ≤ 1% achieved at 6 months) without ever achieving MMR (BCR‐ABL1 IS ≤ 0.1% during the follow‐up period). These patients demonstrated molecular stability but were unable to achieve any further remission.

Fisher's exact tests revealed a statistically significant correlation between prior loss of MMR on imatinib and superior molecular responses to dasatinib (*p* = 0.002), with 61.8% achieving MR4.5. Other response subgroups showed no significant correlations, possibly limited by small sample sizes. However, overall global association (all subgroups combined) was statistically significant (*p* = 0.003).

Twelve patients who did not achieve the standard optimal response (ELN 2024) on low‐dose dasatinib were subjected to dose escalation with standard‐dose for 3 months. Eventually, all 12 patients (22.6%) required a switch to another TKI—nilotinib/ponatinib, in view of sustained failure of response to the standard‐dose dasatinib trial.

Of the five patients who had maintained the previously achieved EMR, but never achieved MMR on dasatinib, two patients developed myeloid blast crisis. Of the four patients who had achieved EMR neither on imatinib nor on dasatinib, one developed myeloid blast crisis, another developed lymphoid blast crisis, and the third one developed T‐lymphoid and myeloid blast mixed phenotype acute leukemia. These patients were managed with appropriate standard chemotherapy regimens and ponatinib.

### 3.3 Kinase Domain Mutation Profiling

**FIGURE 1:**
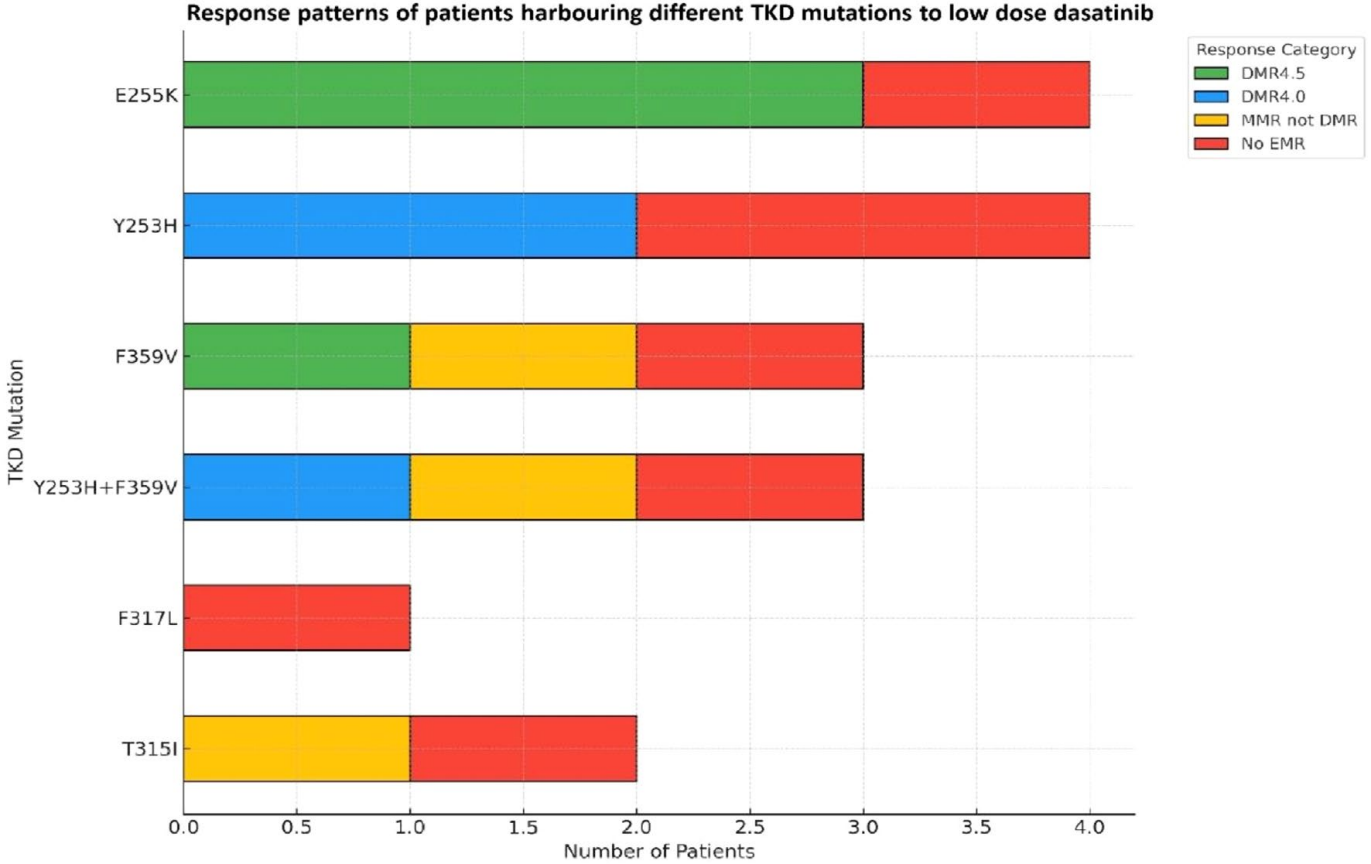
Stacked bar chart showing molecular response patterns by different tyrosine kinase domain (TKD) mutations detected via Sanger sequencing (*n* = 17/53).

TKD mutation analysis done retrospectively in all 53 patients revealed the presence of mutations in 17 patients (32.1%). Of these, 14 patients (26.4%) harbored mutations classified as sensitive to dasatinib, while 3 patients (5.7%) exhibited resistant mutations. Among sensitive mutations, E255K and Y253H were detected in 4 patients (7.5%) each; F359V was seen in 3 patients (5.7%); compound mutations involving both Y253H and F359V were observed in 3 patients (5.7%). Regarding resistant mutations, one patient (1.9%) harbored the F317L mutation while two patients (3.8%) had the T315I mutation. Response patterns to low‐dose dasatinib among our cohort with different TKD mutations are depicted in a stacked bar chart (Figure 1).

### 3.4 Predictors of Response and Progression

Multivariate analysis in our study population showed that the presence of T315I mutation (HR = 3.67, 95% CI = 1.78–7.56, *p* = < 0.001), a high ELTS risk score (HR = 2.12, 95% CI = 1.10–4.09, *p* = 0.025), and baseline BCR‐ABL1 levels > 100% IS (HR = 2.45, 95% CI = 1.18–5.10, *p* = 0.016) independently predicted poor response to dasatinib. Pre‐dasatinib BCR‐ABL1 levels 10%–100% IS showed only marginal association (HR = 1.98, *p* = 0.079). Multivariate analysis for predictors of disease progression to accelerated/blast phase showed that pre‐dasatinib BCR‐ABL1 levels of 10%–100% IS (HR = 2.05, 95% CI = 1.01–4.16, *p* = 0.047) and presence of T315I mutation (HR = 2.98, 95% CI = 1.42–6.25, *p* = 0.004) doubled and tripled the risk, respectively. Factors such as age, gender, duration of response to imatinib, mere presence or absence of KD mutation were not found to have any predictive significance.

### 3.5 Adverse Event Distribution

**FIGURE 2:**
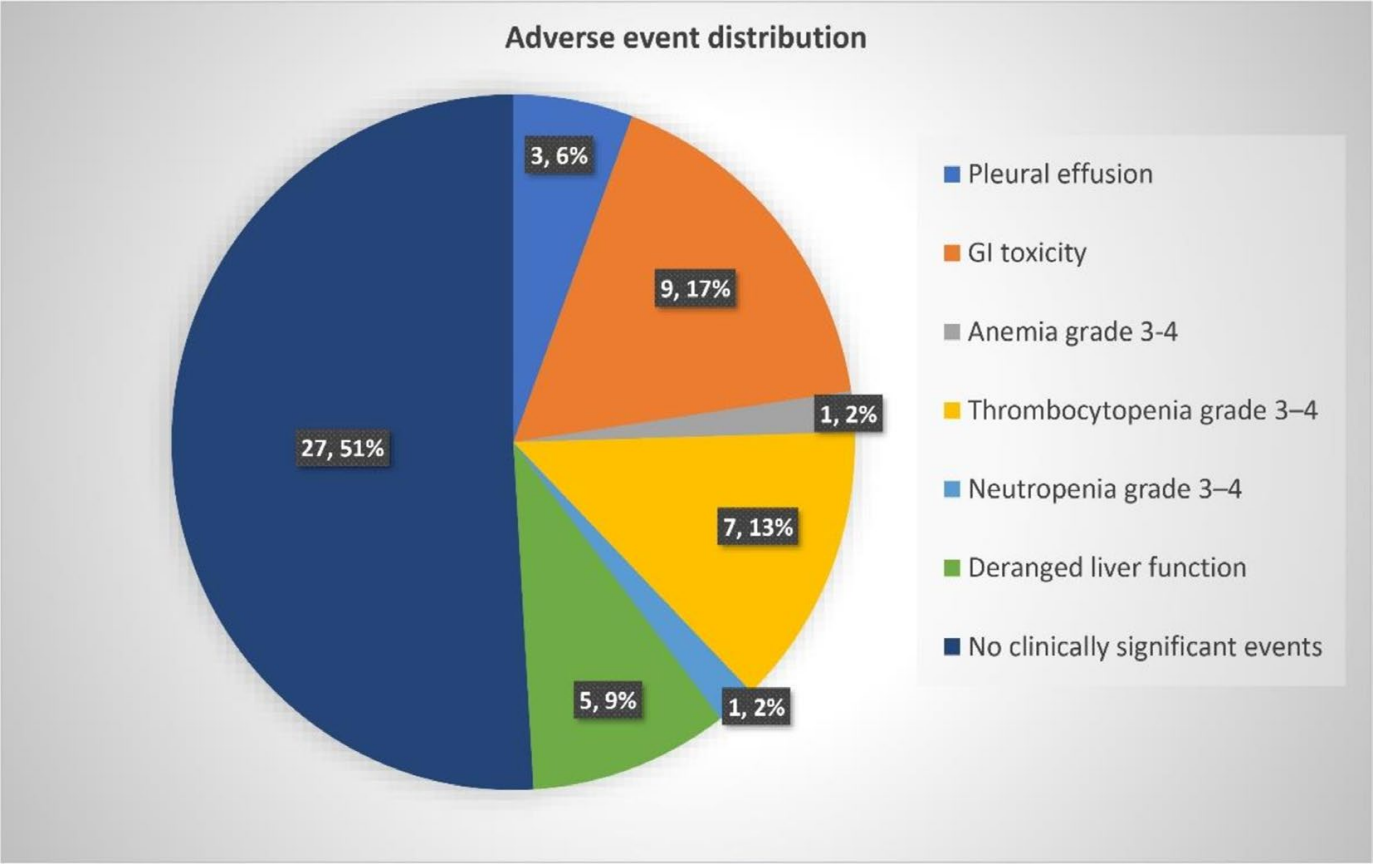
Pie chart summarizing clinically significant adverse event profile of low‐dose dasatinib.

Out of the 53 patients, 26 (49.1%) patients developed clinically significant adverse events on low‐dose dasatinib. The distribution of adverse events among our cohort is depicted in a pie chart (Figure 2).

Two of the patients who had progressed to myeloid blast crisis developed malignant pleural effusion and were managed with standard chemotherapy and ponatinib. One patient with benign moderate pleural effusion also required a switch from dasatinib to nilotinib. Gastrointestinal toxicity was observed in nine patients; three developed dasatinib‐induced colitis requiring a switch to nilotinib, while the remaining six were managed conservatively. One patient who developed grade 3–4 anemia and neutropenia was managed with erythropoietin and G‐CSF, respectively. Patients with grade 3–4 thrombocytopenia were treated with thrombopoietin (TPO) agonist romiplostim. Patients who developed mild to moderate deranged liver function did not necessitate any specific intervention. No cases of deranged renal function/cardiac events were reported.

Therefore, of the 53 patients, only 4 (7.5%) patients (one benign pleural effusion plus three colitis) developed drug intolerance severe enough to require dasatinib discontinuation and TKI switch. Development of malignant pleural effusion in two other patients requiring drug switch was attributed to disease progression and drug failure.

### 3.6 Survival Analyses

**FIGURE 3:**
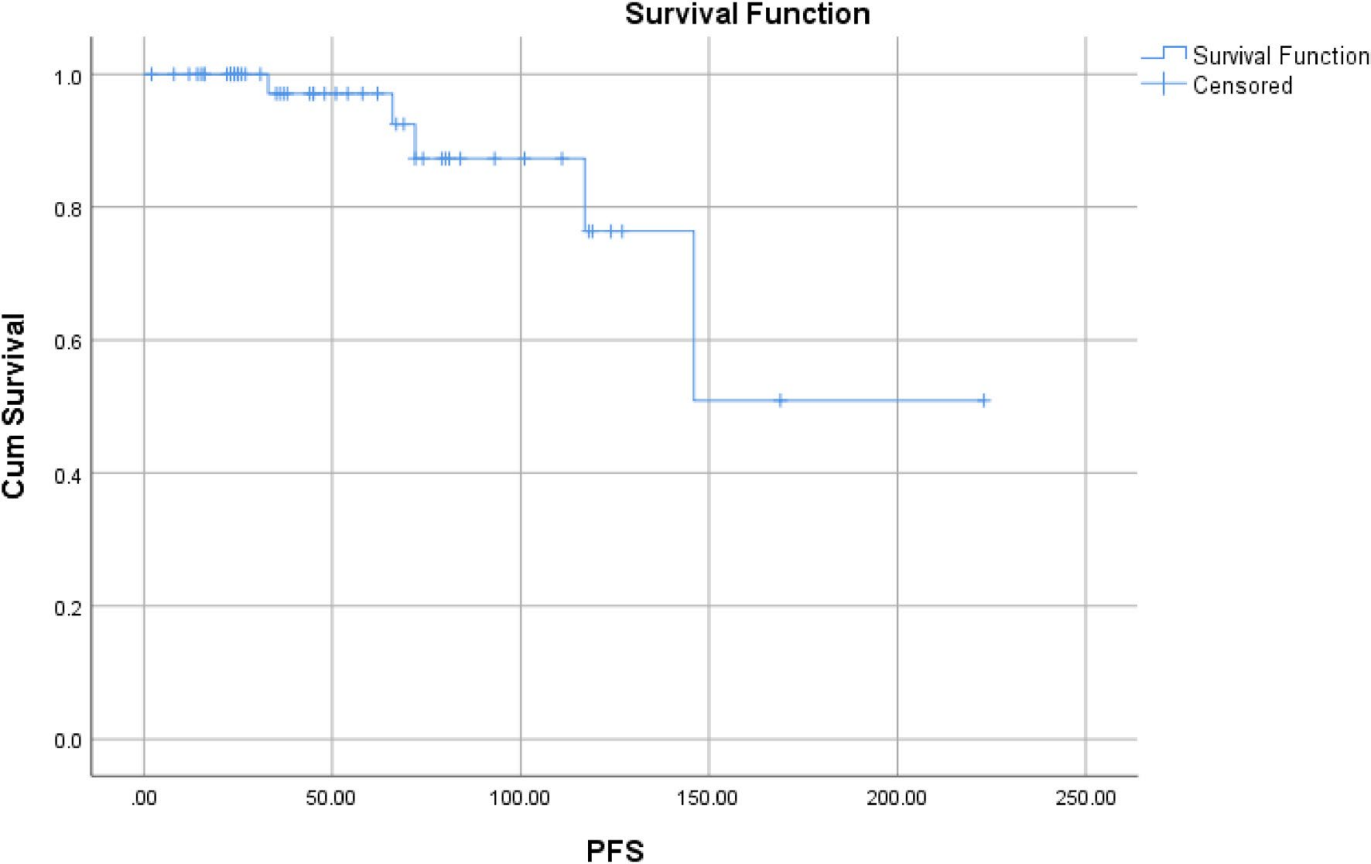
Kaplan–Meier curve illustrating progression‐free survival (PFS) of the study cohort (*n* = 53).

**FIGURE 4:**
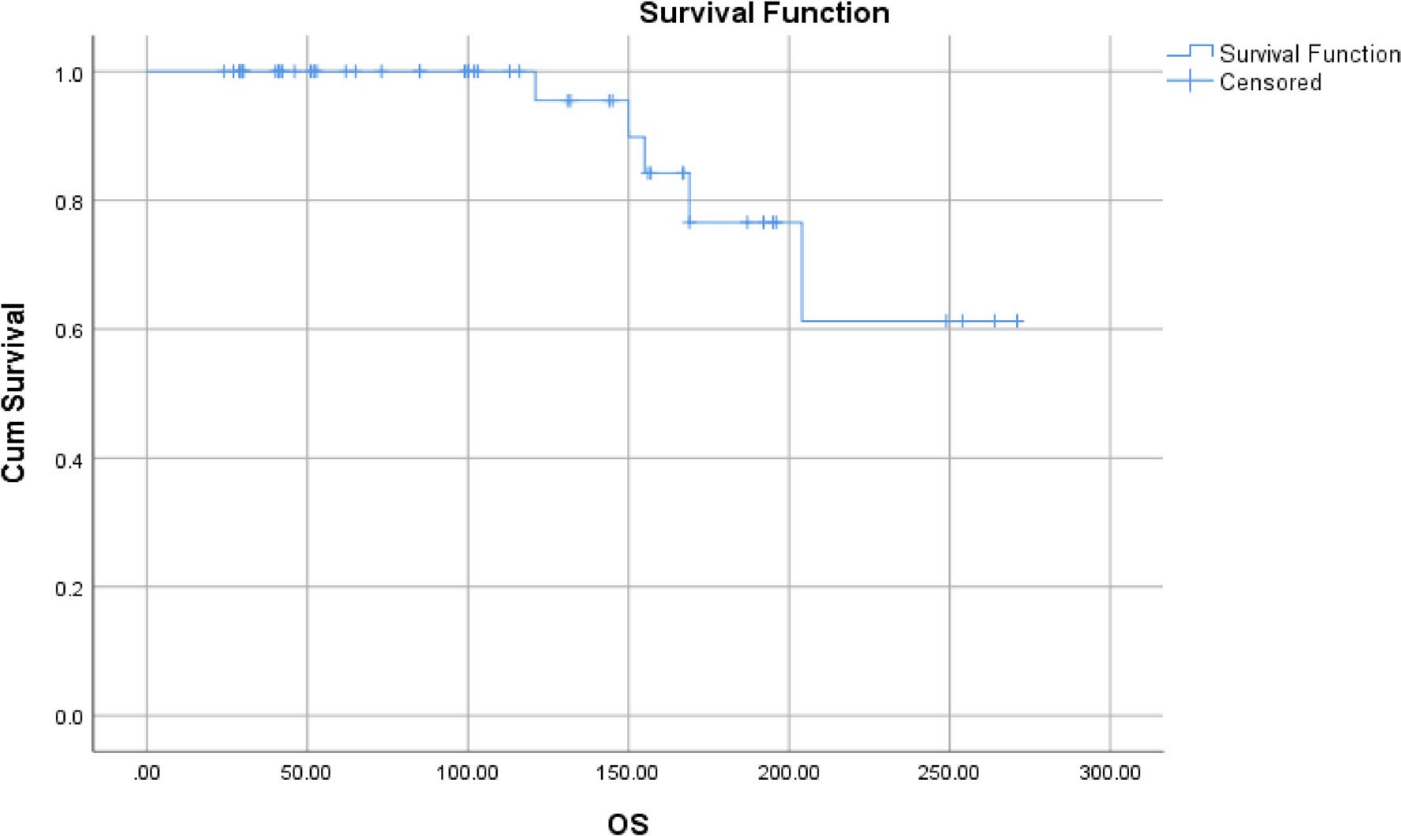
Kaplan–Meier curve illustrating overall survival (OS) of the study cohort (*n* = 53).

In our study cohort, 5 (9.4%) patients progressed to blast crisis during a median period of follow‐up for 93 months (range: 36–146 months). Five (9.4%) patients succumbed to the disease during a median follow‐up of 97 months (range: 69–146 months). The Kaplan–Meier survival estimates for PFS and OS are depicted in Figures 3 and 4, respectively.

Kaplan Meier survival estimates showed that 3‐year PFS in imatinib‐resistant CML‐CP patients treated with low‐dose dasatinib was 100%, 10‐year PFS was 85%, and 20‐year PFS was 50%. The 10‐year OS was 100% and 20‐year OS was 60% in our study cohort.

## 4 Discussion

This retrospective analysis investigates the real‐world efficacy and safety of low‐dose dasatinib (50 mg daily) in patients with CML‐CP who developed resistance to frontline imatinib therapy. Our findings reveal that low‐dose dasatinib is not only effective in achieving DMR but also demonstrates a favorable safety profile with a notably low incidence of adverse events requiring dose modification or TKI switch. These results offer clinically relevant insights for optimizing second‐line therapy in CML, particularly in resource‐limited settings.

While the primary cohort reflects patients managed with dasatinib after imatinib failure, it is important to clarify that the practice of high‐dose imatinib use in CML patients who failed to achieve EMR/MMR, though now discouraged, was historically common in India, particularly in resource‐constrained public institutions prior to 2015 [14]. As reported by Rajan Yadav et al. (*Cureus*), escalation of imatinib to 600–800 mg yielded hematologic responses in over 60% of patients, with a median PFS of 48 months [15]. Although studies suggest superior outcomes with early switch to second‐generation TKIs, financial constraints often dictated continued imatinib‐based strategies during the early part of our 23‐year study period. We have acknowledged this temporal evolution in treatment patterns as a potential confounder in our molecular response rates and TKD mutation analysis. Prolonged exposure to higher doses of imatinib may have facilitated clonal evolution and resistance mechanisms, including kinase domain mutations, thereby influencing subsequent response to dasatinib [16]. This highlights the importance of interpreting our results within the context of evolving guideline‐based care and the real‐world limitations of access to TKIs in low‐ and middle‐income countries.

Among the 53 imatinib‐resistant patients analyzed, 22 (41.5%) achieved MR4.5 and an additional 11 (20.8%) achieved MR4.0 on low‐dose dasatinib. This cumulative 62.3% DMR rate is highly encouraging, particularly considering that only 50 mg of dasatinib was used—a dose significantly lower than the standard 100 mg [8]. The MR4.5 rate of 41.5% in our cohort mirrors the study by Naqvi et al. which demonstrated 46% MR4.5 at 12 months with low‐dose dasatinib in newly diagnosed patients [17], with significantly fewer adverse events as compared to the standard‐dose group but exceeds the DASISION trial's standard‐dose arm which showed 22% MR4.5 at 3 years [18]. This paradox may reflect differences in patient selection: (1) secondary resistance dominance: 64.2% of our cohort had prior MMR loss, a subgroup shown to retain sensitivity to second‐line TKIs; (2) ELTS risk stratification: high‐risk patients (41.5%) in our study achieved lower DMR rates (22.7%), consistent with ELTS as a predictor of TKI response [19]. This finding is consistent with prior studies suggesting that dasatinib at lower doses retains potent BCR‐ABL1 inhibitory activity with reduced toxicity [10, 11, 17].

Our study demonstrates that low‐dose dasatinib is particularly effective in patients who initially achieved but later lost MMR on imatinib. Among these patients (*n* = 34), 61.8% achieved DMR (MR4.5), while 20.6% attained MR4.0, and 17.6% regained MMR without MR (*p* = 0.002). This subgroup likely retains residual imatinib sensitivity and demonstrates favorable biology, suggesting that low‐dose dasatinib is sufficient to recapture durable responses in such cases. This highlights the pivotal role of MMR—not just as a molecular target, but as a real‐world surrogate marker for long‐term disease control. The findings align with the DASISION trial, where standard‐dose dasatinib yielded a 5‐year MMR rate of 76% in newly diagnosed CML patients [20]. Our data suggest that even at half the standard‐dose, dasatinib can induce robust molecular responses in imatinib‐resistant patients with prior MMR. However, the median time to DMR in our cohort is 51 months which is slower than standard‐dose dasatinib (24–36 months) [21, 22], suggesting that prolonged therapy is needed for depth. This subgroup may benefit most from dose optimization, avoiding unnecessary toxicity from higher doses.

In contrast, patients with primary imatinib resistance (*n* = 7) or those who lost both MMR and EMR (*n* = 5) had poorer outcomes, with only 14.3% and 20% achieving MR4.0, respectively (*p* = 0.56 and *p* = 0.29). This is consistent with prior studies indicating that primary resistance correlates with increased probability of BCR‐ABL mutations and inferior TKI responses [5]. Notably, 71.4% of patients who never achieved MMR on imatinib (*n* = 7) maintained only EMR on dasatinib, with two progressing to blast crisis. Poor outcomes (57.1% failure to achieve EMR on low‐dose dasatinib) in patients with primary imatinib resistance are likely due to two reasons: (1) BCR‐ABL1‐independent resistance mechanisms such as upregulation of Lyn kinase or Src pathways, which require alternative targeting and (2) undetected TKD mutations exhibiting resistance to imatinib as well as dasatinib [5, 12, 23].

These patterns underscore the prognostic significance of the type of imatinib failure—primary versus secondary resistance—in shaping subsequent TKI strategies and the need for alternative therapies (e.g., ponatinib) in high‐risk subgroups [23].

The absence of bosutinib and asciminib in our sequencing reflects real‐world limitations in drug access and regulatory timelines during the 23‐year study period. Both agents became available in India only during the latter part of the study, with restricted institutional access. Additionally, in a few patients who did receive bosutinib off‐protocol, intolerance due to gastrointestinal toxicity limited its continued use. Asciminib, although promising, was introduced very late in the study timeline and was not accessible for routine clinical use. Therefore, our post‐dasatinib treatment algorithm predominantly included nilotinib or ponatinib, with the latter reserved for T315I‐mutant cases. This pragmatic sequencing underscores regional disparities in TKI access and illustrates the contextual challenges of implementing guideline‐recommended strategies in resource‐limited settings.

Baseline BCR‐ABL1 levels at diagnosis correlated with response outcomes in our cohort. Patients with transcript levels > 100% IS at baseline showed poor response to treatment, although no evidence of such correlation was reported in the literature. However, the utility of baseline BCR‐ABL levels to identify patients who require a higher dose of imatinib or more potent TKIs warrants further studies with a larger cohort and longer follow‐up [24]. BCR‐ABL1 transcript levels > 10% IS prior to dasatinib initiation in our cohort showed an increased risk of progression to blast phase. This reaffirms prior studies where molecular burden at the time of TKI switch predicted future response [25]. Although BCR‐ABL1 transcript levels are a dynamic marker, their predictive value at TKI transition points cannot be underestimated.

TKD mutation analysis revealed mutations in 32.1% of patients. Several studies report similar frequencies ranging from 20% to 40% in imatinib‐resistant cohorts, detected by Sanger sequencing [12]. Notably, certain mutations such as V299L are known to confer reduced sensitivity to dasatinib [12]. Universal failure of responses in T315I‐mutant cases reinforces the need for mutation profiling before TKI switches. The finding is consistent with that of Nicolini et al., who showed that mutation type, rather than just mutation presence, impacts dasatinib outcomes [26]. Our findings validate the European LeukemiaNet (ELN) recommendation to avoid dasatinib in T315I+ patients. This underscores the importance of mutation‐guided therapy such as an early switch to ponatinib in T315I+ cases, which showed improved outcomes in the OPTIC trial [27].

The relatively younger median age of 50 years in our cohort, compared to the global median of ~64 years for CML diagnosis [2], reflects the population structure in India and the selection bias inherent to a tertiary care referral center that manages predominantly transplant‐eligible and clinically fit patients. Our study focuses on the efficacy and safety of low‐dose dasatinib in imatinib‐resistant CML patients, rather than on age‐stratified outcomes. The observation that younger patients—who may have relatively more aggressive disease—demonstrated high rates of DMR with minimal toxicity supports the potential extrapolation of this approach to older patients, who typically have more indolent disease biology. While comorbidities and pharmacodynamic differences in elderly patients may affect response kinetics, the trend toward effective disease control with reduced toxicity is likely to remain consistent, supporting the generalizability of our findings. Nonetheless, dedicated prospective evaluation in elderly populations would further validate this extrapolation.

Additionally, patients in the high‐risk category by ELTS scoring were significantly more likely to have poorer responses, reinforcing the clinical utility of risk stratification in predicting outcomes and guiding early treatment decisions [19]. Interestingly, traditional factors like age and gender did not significantly influence outcomes in our cohort. This supports the growing consensus that treatment success in CML today hinges more on molecular characteristics and dynamic response milestones than on baseline patient demographics [1, 12].

While our study demonstrates encouraging efficacy and tolerability with low‐dose dasatinib (50 mg daily) in imatinib‐resistant CML‐CP patients, it is important to consider the potential mechanisms contributing to suboptimal response or resistance in a subset of patients. One plausible explanation is clonal selection during prior imatinib therapy, which may enrich for resistant subclones harboring BCR::ABL1 kinase domain mutations (e.g., T315I, F317L) or additional cytogenetic abnormalities, which are less responsive to second‐generation TKIs at lower doses [28, 29]. Additionally, the persistence of leukemia stem cells (LSCs), which are inherently less dependent on BCR::ABL1 signaling and often quiescent, presents a key therapeutic challenge [30, 31]. These stem‐like cells may survive both imatinib and dasatinib therapy, especially at reduced doses, thereby contributing to molecular persistence and eventual resistance [32]. Dasatinib, though more potent than imatinib, may not completely eradicate these primitive clones at lower doses, particularly if bone marrow microenvironmental factors (e.g., niche protection, hypoxia) favor LSC survival [33]. Furthermore, inter‐patient variability in drug metabolism and transporter activity may influence intracellular dasatinib levels, potentially compromising efficacy in some individuals. These factors highlight the need for individualized TKI strategies and underscore the importance of ongoing molecular monitoring to guide timely therapeutic adjustments.

Several prior studies have evaluated low‐dose dasatinib, particularly in the frontline setting. The CA180‐034 study demonstrated the efficacy of standard‐dose dasatinib in imatinib‐resistant CML‐CP, with a 7‐year PFS of 42% and OS of 65% [8]. Our cohort showed a 10‐year PFS of 85% and OS of 100%, which compares favorably despite using only low‐dose dasatinib. This could be attributed to better patient selection, early identification of resistance, and close molecular monitoring. Recent trials including the DESTINY study have highlighted the possibility of dose reduction in sustained responders. Clark et al. showed that dose de‐escalation to 50 mg in patients with stable MR4.0 maintained molecular response in > 90% of cases [34]. Our study extends this concept to imatinib‐resistant patients, suggesting that low‐dose dasatinib is not only viable for maintenance but also for reinduction of response after resistance, especially where long‐term TKI therapy is anticipated. However, five patients progressed to blast crisis: three myeloid, one lymphoid, and one mixed phenotype. Notably, all of these cases occurred in patients who had persistent suboptimal molecular responses on dasatinib and had not achieved MMR and/or EMR. This reinforces that failure to achieve MMR remains a critical inflection point in CML management, predictive of disease transformation.

The tolerability of low‐dose dasatinib in our study cohort was excellent. One previous study using the standard 100 mg once daily dose in imatinib‐resistant CML patients has reported high rates of grade 3–4 neutropenia and/or thrombocytopenia, with up to 52% of patients affected; 17% developed grade 3–4 anemia. The study reported a frequent need for drug interruption (37%) and supportive care [35]. In contrast, our cohort treated with a low dasatinib dose showed a markedly reduced incidence of severe hematologic toxicity potentially due to reduced myelosuppressive effects while maintaining therapeutic efficacy. Among 53 patients, only one developed grade 3–4 anemia and neutropenia, seven patients developed grade 3–4 thrombocytopenia, all of whom were managed conservatively. None of the patients required dose interruptions, highlighting the feasibility of continued treatment without compromising patient safety or requiring frequent dose modifications. The standard approach to TKI‐associated cytopenias includes treatment interruption and reinitiation per prescribing guidelines. However, in our cohort, particularly given the use of low‐dose dasatinib where further dose reduction was not feasible, supportive agents such as ESAs, G‐CSF, and TPO agonists were selectively used off‐label to avoid unnecessary treatment interruption. Importantly, all patients who developed grade 3–4 cytopenias were evaluated with bone marrow studies and peripheral smears to exclude disease progression, accelerated phase, or transformation to blast crisis. In all such cases, there was no evidence of progression, supporting a drug‐related etiology. While this strategy may deviate from guideline‐directed practice, it reflects pragmatic physician decisions in a real‐world, resource‐constrained environment.

Pleural effusion, requiring dasatinib discontinuation, occurred in only three patients, of whom only one (2%) was reported to have benign moderate effusion attributable to drug toxicity. The remaining two cases were reported to be malignant effusions in cases that had progressed to myeloid blast crisis. This is comparable to a 6% occurrence of effusion with the use of frontline low‐dose dasatinib [17]. This is significantly lower than in standard‐dose studies, where pleural effusion of any grade requiring drug discontinuation occurs in up to 28%, supporting dose reduction as a strategy to mitigate effusions while preserving efficacy [36]. In the 7‐year study by Shah et al., standard‐dose dasatinib was associated with gastrointestinal toxicity in 27%–42% of patients, mostly grade 1–2, with grade 3 events in < 5% [8, 37]. Dasatinib‐induced colitis was reported in 1%–10% [38]. In our cohort, 9 patients (17%) developed GI toxicity, including three cases of colitis (5.7%). All colitis cases required permanent discontinuation of dasatinib. This suggests that while GI toxicity is less frequent with lower doses, clinically significant colitis can still occur. No cardiac events were reported; no renal dysfunction was observed, reinforcing dasatinib's renal safety. Liver function abnormalities were mild, requiring no interventions [37]. Overall, the safety profile supports the use of low‐dose dasatinib, with a cumulative incidence of drug discontinuation reported at 7.7%. Drug switches are common in TKI‐treated CML cohorts, and guidelines support individualized sequencing based on toxicity profile, mutation spectrum and treatment goals [12].

To our knowledge, this is one of the largest and most detailed real‐world studies evaluating low‐dose dasatinib exclusively in imatinib‐resistant CML‐CP patients. The homogeneity of the cohort, robust follow‐up, detailed molecular monitoring, and incorporation of mutation analysis strengthen the validity of our conclusions. However, the study has several limitations. First, the retrospective design introduces potential biases related to selection, missing data, and confounding factors. Second, TKD mutation testing was not uniformly available at baseline but was introduced during the course of the study. Third, the sample size within subgroups (e.g., primary resistance) was small, limiting statistical power for subgroup analysis. Finally, the absence of a direct comparator arm (e.g., standard‐dose dasatinib) precludes definitive conclusions regarding relative efficacy.

## 5 Conclusion

Our findings suggest that low‐dose dasatinib is a feasible and effective second‐line option in patients with CML‐CP who are resistant to imatinib, particularly in those with secondary resistance. The favorable safety profile and long‐term outcomes observed in our study support broader use of dose de‐escalation strategies in routine practice, particularly in regions with cost constraints or where drug toxicity is a major concern. Future prospective trials comparing low‐dose versus standard‐dose dasatinib head‐to‐head in imatinib‐resistant patients are warranted.

## Author Contributions

**Nandhini Gangadaran:** conceptualization (equal), data curation (equal), formal analysis (equal), project administration (equal), writing – original draft (lead), writing – review and editing (equal). **Harshal Mamlekar:** conceptualization (equal), data curation (lead), formal analysis (equal), project administration (equal), writing – review and editing (equal). **Souvik Saha:** data curation (lead), formal analysis (equal), writing – review and editing (equal). **Rajesh Kashyap:** conceptualization (equal), project administration (equal), supervision (equal), writing – review and editing (equal). **Sanjeev Yadav:** conceptualization (equal), supervision (equal), writing – review and editing (supporting). **Khaliqur Rahman:** validation (equal). **Ruchi Gupta:** validation (equal). **Mona Vijayaran:** validation (equal). **Manish Singh:** validation (equal). **Dinesh Chandra:** validation (equal).

## Ethics Statement

The study protocol was reviewed and approved by the Institutional Ethics Committee, ensuring adherence to ethical guidelines for retrospective data analysis and patient confidentiality.

## Conflicts of Interest

The authors declare no conflicts of interest.

## Data Availability

The data that support the findings of this study are available from the corresponding author upon reasonable request.
